# Biocompatibility Testing of Liquid Metal as an Interconnection Material for Flexible Implant Technology

**DOI:** 10.3390/nano11123251

**Published:** 2021-11-30

**Authors:** Katharina Foremny, Steven Nagels, Michaela Kreienmeyer, Theodor Doll, Wim Deferme

**Affiliations:** 1 ORL Department, Hannover Medical School, Carl-Neuberg-Straße 1, 30625 Hannover, Germany; kreienmeyer.michaela@mh-hannover.de (M.K.); doll.theodor@mh-hannover.de (T.D.); 2 Cluster of Excellence Hearing4All, Carl-Neuberg-Straße 1, 30625 Hannover, Germany; 3 Institute for Materials Research (IMO), Hasselt University, Wetenschapspark 1, 3590 Diepenbeek, Belgium; steven.nagels@uhasselt.be (S.N.); wim.deferme@uhasselt.be (W.D.); 4 IMEC vzw, Division IMOMEC, Wetenschapspark 1, 3590 Diepenbeek, Belgium

**Keywords:** galinstan, silicone rubber, sterilization, biocompatibility, bacteria, in-vivo

## Abstract

Galinstan, a liquid metal at room temperature, is a promising material for use in flexible electronics. Since it has been successfully integrated in devices for external use, e.g., as stretchable electronic skin in tactile sensation, the possibility of using galinstan for flexible implant technology comes to mind. Usage of liquid metals in a flexible implant would reduce the risk of broken conductive pathways in the implants and therefore reduce the possibility of implant failure. However, the biocompatibility of the liquid metal under study, i.e., galinstan, has not been proven in state-of-the-art literature. Therefore, in this paper, a material combination of galinstan and silicone rubber is under investigation regarding the success of sterilization methods and to establish biocompatibility testing for an in vivo application. First cell biocompatibility tests (WST-1 assays) and cell toxicity tests (LDH assays) show promising results regarding biocompatibility. This work paves the way towards the successful integration of stretchable devices using liquid metals embedded in a silicone rubber encapsulant for flexible surface electro-cortical grid arrays and other flexible implants.

## 1. Introduction

Flexible surface electro-cortical grid arrays (ECoG) are a valuable alternative to penetrating multi-electrode arrays (MEA). Their usability in both acute and chronic recordings is under investigation for numerous material combinations [1,2,3,4] and their potency for good recordings has been shown as well [2,3]. Yet they are limited in their flexibility by the rigid materials used for their conductive pathways. Several methods to improve flexible electronics are under investigation [5], including the usage of rigid conductors with improved geometric patterning techniques [6,7], polymers containing conductive (nano)particles, e.g., carbon nanotubes [8,9] and liquid metals [5,10].

A common denominator in novel compliant interconnections is their deep roots in materials science. Gupta et al. demonstrate tremendous increments in flexibility by thinning down metallic structures [11]. By patterning thin metals into a 2D waveform, flexibility is converted into stretchability [12]. Sun et al. further leverage out-of-plane deformation to accommodate increased stretch [13], whereas coping with extreme strain percentages in stretch (approx. 1000%) was achieved via drastically miniaturized wavy metallic structures [14]. Their practical application however seems limited to chip-level. Conductive fillers were blended into an elastomeric matrix as an alternative approach to achieve compliant interconnections [15], and the influence of particle size, shape and composition in the pursuit of highest conductivity at lowest filler concentration was studied [16]. However, due to its composite nature, this approach can never attain conductivities neighboring the order of magnitude of bulk metals.

Since its introduction by Dickey and co-workers in 2008, gallium-based liquid metal alloys have gained tremendous popularity for the creation of compliant interconnections [17], and recent advances on new alloys were made [18]. A conductivity for liquid metal just one order of magnitude below bulk copper was reported [19] and an indication of their safety in handling—even in in vivo studies—have proved successful [20]. Ladd et al. show that a stabilizing oxide layer allows for thin elongated patterns of liquid metals that normally would be impossible under the influence of surface tension [21]. Strains up to 700% for a silicone microchannel filled with liquid metal were reported [22]. Its failure is mainly bound by the mechanical limits of the silicone encapsulant.

The possibility of printing liquid metals [4] might be a flexible solution to implement the use of liquid metals in the implant manufacturing process. Many research papers focus on patterning, since this is notoriously difficult [23]. Neumann et al. investigated aerosol printing of the liquid metal and the elastomeric coating for rapid production of stretchable electronics [24]. In addition, stencil printing was recently acquired for the deposition of 200 µm width liquid metal traces, acting as an interconnect [25]. The tapping method [26], inkjet printing [27], direct wetting [28] and selective wetting [29] were applied for the deposition of these liquid metals.

As already announced by Dickey et al., “liquid metals provide the best combination of conductivity and deformability” [10]. This feature is our reason for evaluating the usability of galinstan, not only as a promising alternative in electronics, but also to further improve medical products, such as ECoGs that are implanted into the human body. Attaching galinstan-based electronic circuits encased in silicone rubber to the skin has been already successfully done [30] and new research shows promising steps towards full integration as electronic skin [31].

A first step towards the usability of a material in medical applications, especially for implants, is to ensure the sterility of the product, hence the destruction or removal of any microorganisms [32]. The relevance of this work lies exactly in this: to evaluate three common sterilization techniques in their application to galinstan liquid metal, paving the road further for using the material in challenging medical applications. The gold standard of sterilization processes is steam sterilization [32] at 121 °C for at least 20 min [33]. This technique is an effective, simple treatment without toxic residue [34]. Since the effect of moist heat on the galinstan samples is non-negligible surface oxidation, sterilization with UV-rays was additionally investigated, even though UV-light is mostly useful for surface sterilization and its efficiency varies between many characteristics. These include the materials and their UV transparency [35], the microorganisms as well as UV wavelength and exposure time, both also having an impact on the material properties of the sample [34]. As a third option, ethanol sterilization was chosen. Storing the sample in 70% ethanol is a low-cost alternative and temperature-independent [34], as well as a method widely used for in-vitro studies [36]. This makes it an interesting alternative to both methods mentioned above; however, depending on the storage time in ethanol, silicone rubber swelling and galinstan dewetting might occur. Even though galinstan would be used as an alternative circuit material without direct tissue contact, the biocompatibility of the materials needs to be established to ensure safety in case of leakage. If leaks occur, the biocompatibility of galinstan itself comes into scope quickly. Save usage in in vivo and in vitro tests has been assumed in many studies. Yet, as Yan et al. already mentioned, no toxicology profiles were found in 2018 [37]. Another study by Guo et al. also shows positive results in biocompatibility tests [38]. This seems to remain the case thus far.

In this work, we present a first study on the usability of UV sterilization, ethanol sterilization and steam sterilization for galinstan samples. Biocompatibility tests using fibroblast cells were conducted directly on galinstan samples to evaluate the sterilization methods. For cell proliferation and morphology evaluation, tests were performed using sample-conditioned cell-culture media. Biocompatibility tests (WST-1 assay) as well as cell toxicity tests (LDH assays) were performed.

## 2. Materials and Methods

### 2.1. Sample Preparation

**Figure 1:**
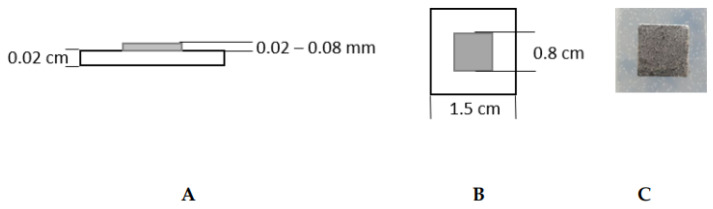
Schematic of the samples prepared with silicone rubber (white) and galinstan (grey). (**A**) Cross section of sample showing the material height; (**B**) top view of sample showing the material width; (**C**) microscopic image of galinstan square on silicone–rubber substrate.

Samples used for sterilization tests were 1.5 cm × 1.5 cm in size and consisted of silicone rubber (Ecoflex 00–30, Smooth-On, Macungie, PA, USA) as substrate material for a galinstan (composition: 68% gallium, 22% indium, 10% tin; Eugen Mueller, Wolframs-Eschenbach, Germany) square as shown in Figure 1. The galinstan-silicone rubber samples were prepared by depositing a 1 mm layer of silicone rubber on a 45 mm × 45 mm carrier substrate of 3 mm thick acrylic. On top of the silicone rubber, a stencil sticker was laser-cut in place with 9 square openings of 8 mm × 8 mm, evenly spaced across the 45 mm × 45 mm surface. Liquid metal was thereafter sprayed over the surface in an even layer of 20 to 50 µm thickness. By removing the stencil sticker, 8 mm × 8 mm squares of liquid metal were left on the surface. The 45 mm × 45 mm silicone rubber sheet underneath the 9 squares of galinstan was subsequently cut into 15 mm × 15 mm squares with a scalpel. Nine squares of 15 mm × 15 mm silicone rubber, each with an 8 mm × 8 mm liquid metal square sitting on top, were used for biocompatibility testing. Silicone rubber-only samples, i.e., without the galinstan, were prepared with the same methods for the same measurements.

### 2.2. Sterilization Methods

The sterilization methods under investigation for galinstan–silicone rubber samples were ultraviolet light (UV; wavelength 254 nm) sterilization, sterilization using 70% (*v*/*v*) ethanol solution, and steam sterilization at 121 °C for 20 min. Two sample types were evaluated. The first sample type consisted of only silicone rubber and the second sample type consisted of silicone rubber and galinstan (see Figure 1). Samples were transferred to a glass petri dish (Ø = 4 cm, Petrischalen Kalk-Soda-Glas 60 mm × 15 mm, Omnilab, Bremen, Germany) for sterilization and first cell-culture tests. The samples were sterilized using UV light for 30 min, 60 min or 90 min. For ethanol sterilization the samples were covered with 70% ethanol for 60 min. Steam sterilization was performed using a Laboklav 8 MSLV (Nr. 08094823/62741, SHP Steriltechnik AG, Haldensleben Germany). A total of 150,000 NIH mouse fibroblast cells/mL were seeded onto each sterilized sample and incubated at 37 °C and 5% CO_2_ for 24 h to evaluate the sterilization success. Cell proliferation and morphology were evaluated by analyzing the cells that grew next to the sample. For imaging, an inverse transmitted-light microscope was used (CKX41, Olympus, Tokyo, Japan).

### 2.3. Biocompatibility Tests

**Figure 2:**
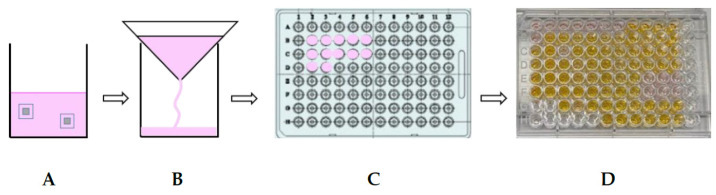
Protocol established for preparation of conditioned cell-culture media for further testing. (**A**) Conditioning of cell-culture media (pink) with two galinstan samples; (**B**) filtration of conditioned media; (**C**) adding the conditioned media to the cells in well plate; (**D**) color change in well plate after adding the WST-1 agent to the media right before measurement.

Due to the material properties of galinstan it is not possible to evaluate cell proliferation and morphology on the sample surface. After first cell tests for sterilization evaluation, it was decided to use cell-culture media conditioned with the samples for the biocompatibility tests, instead of growing cells directly on the material. This method is an alternative to cell growth on the sample suggested in ISO 10993-5. A protocol to condition cell-culture media with galinstan–silicone rubber samples and silicone rubber samples was established. Figure 2 shows a schematic of this protocol. Two samples made of silicone rubber or galinstan–silicone rubber were transferred to a glass petri dish or a 100 mL Schott glass bottle (Schott, Mainz, Germany). The samples were sterilized in the petri dish using UV sterilization for 90 min or in the glass bottle using steam sterilization, followed by drying at 45 °C for two days. Twenty millilitres of cell-culture medium were added to sterilized samples after transferring UV light sterilized samples into a 100 mL Schott glass bottle under sterile conditions. As cell-culture medium, Dulbecco’s modified eagle medium without fetal calf serum (FCS, Biochrom, S0615, Berlin, Germany) and phenol red (DMEM; F0475, Biochrom, Germany) were used. A total of 0.5% L-Glutamine solution (K0282, Biochrom, Berlin, Germany) was added. Samples and cell-culture media were incubated at 37 °C and 5% CO_2_ for seven days. The media were subsequently filtered using a high-purity filter paper for qualitative analysis (Hahnemühle, Dassel, Germany) to ensure that samples and detached particles were removed. Ten percent FCS was added to both media after preparing 5 mL aliquots. Media samples were frozen at −20 °C until testing. All following cell-culture tests were performed using the conditioned media.

For the biocompatibility evaluation, a water-soluble tetrazolium dye assay (WST-1 assay) and a lactate dehydrogenase assay (LDH assay) were performed. Using the WST-1 assay, information concerning the cell metabolism is gained and through their metabolism performance the biocompatibility can be determined. The LDH assay shows the biotoxicity of samples by measuring the amount of lactate dehydrogenase in the media via a reaction with the reaction agent. Lactate dehydrogenase (LDH) is released into the media when plasma membranes are damaged. Its presence therefore indicates cell damage.

To perform the WST-1 assay, conditioned media were pipetted into wells of a 96-well plate (PS 96 well sterile TC, Costar, (Corning, NY, USA). Unconditioned DMEM was used as negative control (cell growth under normal cell-culture conditions) and DMSO (A994.1, Roth, Germany) was used as positive control (cells are killed). A total of 100 µL of control media or conditioned media were added to the wells. A total of 10,000 NIH mouse fibroblast cells/mL were added to the media. Cells were incubated for 48 h under cell-culture conditions at 37 °C and 5% CO_2_. Before adding the cell proliferation reagent WST-1 into the wells with incubated cells, a light microscopic inspection was carried out. WST-1 reagent (WST-1 cell proliferation assay, Roche, Basel, Switzerland) was added according to the data sheet instructions and the cells were incubated for an additional 40 min. Colorimetric measurements were done using a plate reader at 450 nm vs. a 650 nm reference. For each sample, six wells were evaluated. To evaluate the biocompatibility, the results from the negative control were set at 100% biocompatibility. The test was repeated three times.

To perform the LDH assay, 10,000 NIH mouse fibroblast cells/mL were seeded into wells of a 96-well plate and incubated for 48 h with either conditioned media or unconditioned DMEM as negative control under cell-culture conditions. As positive control 10% Triton X (Sigma-Aldrich, Art. Nr. X100, St. Louis, MO, USA) in PBS (Gibco, ThermoFisher Scientific, Waltham, MA, USA) was used to perforate the cell’s plasma membrane, resulting in cell death and a high LDH level in cell-culture media. A light microscopic inspection was carried out before the reaction mixture for LDH was added. Fifty microliters of each sample medium were transferred to a 96-well flat bottom plate. Then 50 µL of reaction mixture (LDH cytotoxicity assay Kit, Pierce^TM^, Waltham, MA, USA) were added and the cells were incubated for 30 min at room temperature, while protected from light. After adding stop solution to the wells, the colorimetric measurements were performed using a plate reader at 450 nm vs. a 650 nm reference. The results from the positive control were set at 100% cell toxicity. For each sample six wells were evaluated. This test was only conducted once.

## 3. Results and Discussion

### 3.1. Sterilization Methods

**Figure 3:**
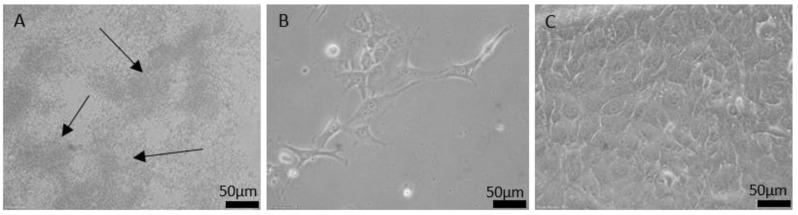
Light microscopic images of cell growth in petri dish after 24 h incubation with (**A**) UV-sterilized, (**B**) ethanol-sterilized and (**C**) steam-sterilized galinstan–silicone rubber sample. In (**A**) mostly bacteria clouds are visible. Image (**B**) shows a low number of fibroblast cells as well as necrotic cells. Image (**C**) shows a confluent fibroblast layer.

Three sterilization methods were compared for usage on galinstan samples. In the petri dishes containing the samples sterilized for 30 min and 60 min under UV light, the cell culture showed extensive bacterial infestation (see Figure 3A). Cell proliferation and morphology could not be evaluated for most samples, due to the bacterial overgrowth. This failure to sterilize may be due to the poor penetration depth of UV light [39] especially for such non-transparent materials as galinstan. Some studies have shown that 1 h UV-light exposure should be long enough to eliminate the signs of infection [36], while in other studies success has been shown to depend also on the UV wavelength used, as well as the kind of microorganisms inhabiting the samples [34]. The exact reason for failure is therefore unclear.

Both sample types sterilized by storing them in ethanol for 60 min showed poor cell proliferation, and necrotic cells in the petri dish next to the sample (see Figure 3B). No bacterial infestation could be detected in the petri dish containing the ethanol sterilized samples. Yet this sterilization method was not further investigated, due to reduced cell growth that is possibly a result of ethanol residue. Moreover, the ethanol sterilization limitation in microorganism destruction is another reason to abandon this method [34].

Using steam sterilization, samples showed no bacterial infestation in cell culture after 24 h. Cells grew into a confluent cell layer; hence proliferation and morphology were as expected from fibroblast cells after 24 h (see Figure 3C). The results were as expected from the gold standard sterilization technique, which allows for sterilization of complex structure due to the steam [32].

**Figure 4:**
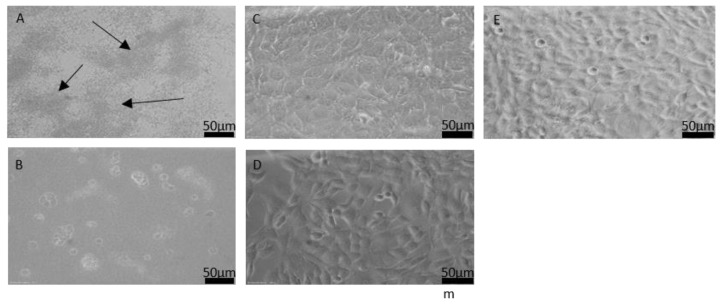
Light microscopic images of cell growth after 48 h incubation in media conditioned with (**A**) galinstan-silicone rubber, (**B**) silicone rubber sample sterilized for 90 min using UV light, (**C**) galinstan-silicone rubber, and (**D**) silicone rubber sterilized using steam sterilization. (**E**) Cells without sample, grown under normal cell-culture conditions. (**A**) The arrows show the bacteria clouds; (**B**) reduced cell growth and necrotic cells on a layer of bacteria; (**C**–**E**) a confluent layer of fibroblast cells.

Conditioning the media with sterilized samples proved doable. Filtration removed particles that had been removed from the samples. The evaluation of cell morphology and proliferation, influenced by the tested samples, was made possible using the conditioned media. After the unsuccessful UV sterilization for 30 min and 60 min, a last attempt was made using UV sterilization for 90 min. A first test showed a cell culture without bacterial contamination. Yet further tests were unsuccessful. The medium conditioned with 90-min UV-sterilized silicone rubber samples was slightly opaque after conditioning. The medium conditioned with galinstan first showed no signs of bacteria. Cells were seeded into well plates with the conditioned media to evaluate cell growth. Tests for both samples had to be discontinued due to bacterial infestation after cell incubation for 24 h (see Figure 4A,B). The following tests using media conditioned with steam-sterilized samples were successful and showed no signs of bacteria in the cell proliferation and morphology tests (see Figure 4C,D). Cells grew to a confluent layer and showed a good morphology. Comparison between these cells and the control well showed no differences in cell growth. Therefore, this sterilization method was subsequently used for the biocompatibility evaluation.

### 3.2. Biocompatibility Tests

**Figure 5:**
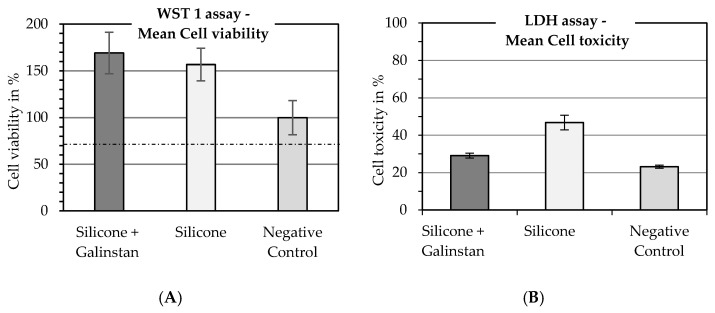
Results of biocompatibility tests using conditioned media. (**A**) Cell viability of galinstan–silicone rubber and silicone rubber measured with the WST-1 assay; N = 18; dotted line is at 70%; (**B**) cell toxicity of galinstan–silicone rubber and silicone rubber measured with LDH assay; N = 6.

For biocompatibility tests, only steam-sterilized samples were used, since the other sterilization methods were eliminated during the sterilization evaluation. It was found after comparison of sterilization methods that steam sterilization is the most reliable sterilization method in combination with the liquid metal galinstan in our case (see Figure 4C,D). Figure 5 shows the cell viability evaluation (WST-1 assay) and cell toxicity evaluation (LDH assay) for the media that were conditioned with the samples for seven days. The results for the negative control are set at 100% cell viability for the WST-1 assay. The results for conditioned media are significantly higher than 100%, indicating an even higher metabolization of the WST-1 reagent in the wells holding the cells cultured with conditioned media, than in the control well. Therefore, it can be assumed that cell growth and metabolism were very good for both silicone rubber and galinstan + silicone rubber. These results are comparable with Guo et al., who showed cell viability over 100% as well [38], and better than the cell survival rates of around 100% reported by Wang et al. [40]. The LDH assay that is used to evaluate cell cytotoxicity showed promising results for galinstan and silicone rubber as well, as can be seen in Figure 5. The results for the positive control are set at 100% cell toxicity. The negative control contains cells that were grown under normal cell-culture conditions using DMEM. Cell toxicity for these cells is at 22%. The cytotoxicity of the media conditioned with galinstan-silicone rubber is below 30% and only slightly higher than the results for the negative control. Results for silicone rubber were slightly more toxic. This stands in contrast to the results for galinstan-silicone rubber, which contains the same silicone rubber as the pure silicone rubber sample. It also stands in contrast to the very high cell viability measured in the WST-1 assay. Several factors can influence the results of such tests. One factor is the amount of repetitions to eliminate rogue results. Since the cell-culture conditions were identical for all samples, a contamination of the cell-culture wells or of the silicone-rubber samples might be possible. Further testing could therefore eliminate this discrepancy. Overall the WST-1 assay and the LDH assay represent only a first biocompatibility evaluation, which needs to be extended further to evaluate the suitability of the material in longer-term implanted devices.

## 4. Conclusions

To evaluate the usability of the liquid metal galinstan, standard sterilization methods were evaluated and first biocompatibility assays performed in accordance with ISO 10993. As far as the authors could find, this was the first evaluation of sterilization processes for galinstan samples. UV sterilization and ethanol sterilization were found to be either unreliable (bacterial infestation) or leading to low cell proliferation on the sample. Steam sterilization, the already established gold standard, was identified as the most reliable sterilization method out of three for galinstan samples. The samples were free of bacteria and the steam sterilization also allows for easy sample handling. For the sterilization processes, the biocompatibility of galinstan was always assumed in the literature, but with no results for biocompatibility in vivo under the guidelines of ISO 10993. Cell growth on galinstan was problematic due to its liquid state. Therefore, a protocol to condition the cell-culture media with the samples was successfully established. First WST-1 assays and LDH assays done using the galinstan-conditioned media suggest a good biocompatibility of galinstan–silicone rubber. These positive sterilization and biocompatibility results pave the way into in-vivo applications, such as flexible patient-individual ECoGs.

## Data Availability

The data presented in this study are available upon request from the corresponding author.
